# Perceptions and attitudes of pediatricians and families with regard to pediatric medication errors at home

**DOI:** 10.1186/s12887-023-04106-x

**Published:** 2023-07-31

**Authors:** Javier González de Dios, Adriana López-Pineda, Gema Mira-Perceval Juan, Pedro J. Alcalá Minagorre, Mercedes Guilabert, Virtudes Pérez-Jover, Irene Carrillo, José Joaquín Mira

**Affiliations:** 1 Pharmacology, Pediatrics and Organic Chemistry, Miguel Hernandez University, San Juan de Alicante, Spain; 2 Paediatrics Department, General University Hospital of Alicante, Alicante, Spain; 3 Institute of Health and Biomedical Research of Alicante, Alicante Spain General University Hospital of Alicante, Alicante, Spain; 4 Clinical Medicine Department, Miguel Hernández University, San Juan de Alicante, Spain; 5 Atenea Research Group, Foundation for the Promotion of Health and Biomedical Research, San Juan de Alicante, Spain; 6 Network for Research on Chronicity, Primary Care, and Health Promotion (RICAPPS), San Juan de Alicante, Spain; 7 Primary Health Centre San Vicente Raspeig I, San Vicente Raspeig, Alicante, Spain; 8 Health Psychology Department, Miguel Hernandez University, Elche, Spain; 9 Alicante-Sant Joan d’Alacant Health Department, San Juan de Alicante, Spain

**Keywords:** Medication errors, Pediatricians, Child, Caregivers, Parents

## Abstract

**Purpose:**

This study aimed to identify the perceptions and attitudes of pediatricians and parents/caregivers regarding medication errors at home, and to compare the findings from the two populations.

**Methods:**

This was a cross-sectional survey study. We designed a survey for working pediatricians and another one for parents or caregivers of children aged 14 years and younger. The survey’s questions were designed to assess provider and parental opinions about the difficulty faced by parents providing medical treatment, specific questions on medication errors, and on a possible intervention program aimed at preventing pediatric medication errors. Pediatrician and parent responses to matching questions in both surveys were compared.

**Results:**

The surveys were administered in Spain from 2019 to 2021. In total, 182 pediatricians and 194 families took part. Most pediatricians (62.6%) and families (79.3%) considered that managing medical treatment was not among the main difficulties faced by parents in caring for their children. While 79.1% of pediatricians thought that parents consulted the internet to resolve doubts regarding the health of their children, most families (81.1%) said they consulted healthcare professionals. Lack of knowledge among parents and caregivers was one of the causes of medication errors most frequently mentioned by both pediatricians and parents. Most pediatricians (95.1%) said they would recommend a program designed to prevent errors at home.

**Conclusions:**

Pediatricians and families think that medical treatment is not among the main difficulties faced by parents in caring for their children. Most pediatricians said they would recommend a medication error reporting and learning system designed for families of their patients to prevent medication errors that might occur in the home environment.

**Supplementary Information:**

The online version contains supplementary material available at 10.1186/s12887-023-04106-x.

## What is known

- Paediatric patients are at higher risk of harm resulting from medication errors than adult patients.
- At home, parents and/or caregivers may make medication errors related to dose, time administration, preparation, drug interaction among other.
- The age, sex and the mother tongue of the caregiver, medical prescriptions including more than two drugs, and certain types of dosing devices increase the risk of error medication made by parents/caregivers.

## What is new

- While pediatricians believe that parents use the internet as their main source of information for resolving doubts related to their children’s health, parents said they consult a healthcare professional.
- Both pediatricians and parents think that the lack of knowledge of parents’ and caregivers’ may be one of the main causes of medication errors at home.
- Pediatricians would recommend a program designed to prevent these errors to the families of their patients.

## Introduction

Medication-related deaths and adverse effects are mainly due to medication errors [1]. Drug safety is particularly important in pediatric patients, as they may be at higher risk of harm resulting from errors than adult patients [2]. Previous studies have uncovered errors at all stages of pediatric medication use, from prescription to administration [3], in the hospital, outpatient and home setting [4].

A 2020 meta-analysis by Gates et al. [4] obtained a rate of medication error in hospitalized children of 15.0 (3.1–49.4) per 100 prescriptions. The authors observed that most medication errors in the hospital setting occurred during prescribing process. In 2019, Glick et al. [5] found that 38% of parents or legal guardians committed some type of error when administering medication to their children in the first two weeks after discharge. Data from the US National Poison Database System showed a mean annual rate of out-of-hospital medication errors in the decade between 2002 and 2012 of 26.42 per 10,000 children aged under 6 years [6]. This should be taken as an approximated value because this system only captures cases reported to poison control center. The medication errors without recognition or errors that were noticed but not reported because individuals involved seek information from other sources were not counted.

Although most studies on pediatric medication errors at home focus on dose errors [5, 7], the literature has shown that parents and/or caregivers also make other mistakes, such as incorrectly preparing or reconstituting the medication [8, 9] or administering the dose twice [6]. Berthe-Aucejo et al. [9] found that among parents and caregivers, risk factors for committing errors when administering medication to pediatric patients included being young, male, and non-native to their country of residence. Higher frequency of errors may also be associated with medical prescriptions that include more than two drugs, and certain types of dosing devices [10]. According to previous studies, analgesics are most commonly involved in pediatric medication errors at home [6, 11].

To reduce the occurrence of these errors, it is necessary to promote the safe use of medication at home. One prevention strategy involves medication error reporting systems [12]; however, as far as we know, no such system exists for the parents or caregivers of pediatric patients [13]. Currently, there is a great deal of interest in how patients and families can report patient safety events and work with policymakers and healthcare professionals to improve patient safety. In fact, in the United States, as well as in other countries such as Finland and Norway, there are initiatives to create independent agencies that can receive reports on adverse events in patient safety, analyze data, identify patterns of harm risks, investigate, and establish prevention and recommendation strategies to prevent adverse events in the home, with a particular focus on medication errors. This study aims to provide a preliminary understanding of the challenges faced by caregivers of pediatric patients in home care, with a specific focus on medication errors, and to identify potential strategies to prevent and minimize them. The goal is to learn from these experiences and prevent future occurrences of such errors. These and other prevention programs should be based on the needs of the users, i.e. pediatricians and parents or caregivers. Thus, the research question was: what are the perceptions and attitudes of pediatricians and families towards pediatric medication errors at home? This study aims to identify and compare the perceptions and attitudes of pediatricians and those of parents/caregivers regarding pediatric medication errors at home.

## Materials and methods

We carried out a cross-sectional survey study spanning the period between November 2019 and March 2021. The study protocol was approved by the Responsible Research Office of Miguel Hernandez University in Elche, Spain (Reference: DPS.MGM.01.19). The patients/participants provided their written informed consent to participate in this study.

Our study included two populations: pediatricians working in public or private healthcare centers in Spain, and parents of children aged 14 years and younger residing in Spain. The inclusion criteria required that all study participants understand the Spanish language, have internet access, participate in the study voluntarily, and sign an informed consent form. Respondents were selected by snowball sampling. Starting with target populations of 12,000 pediatricians working in Spain and 5,000,000 families with children living in Spain, and applying a confidence level of 95% and a margin of error of 8%, we established a minimum required sample size of 149 pediatricians and 151 families.

After performing a literature review and consulting with two pediatricians, we designed a survey for pediatricians and another for families. The family survey underwent a legibility assessment and was initially presented to two families to ensure comprehensibility. The survey for pediatricians included nine multiple-choice questions and one open question (Supplementary Document 1), and the survey for families included 11 open questions and three multiple-choice questions (Supplementary Document 2). Questions on general difficulties faced by parents in caring for their children - like feeding concerns, and symptoms of disease among others-, were included to obtain the initial (spontaneous) opinion of the respondents about the difficulties of medical treatment. The questions in both surveys were grouped into three thematic blocks: difficulties faced by parents in caring for their children, pediatric medication errors at home, and prevention strategies. In addition, the following sociodemographic variables were collected from pediatricians: sex, age, years of experience, specialty, level of care, and Spanish autonomous community of residence; and from parents: sex, age, marital status, educational attainment, Spanish autonomous community of residence, number of children, number of emergency room (ER) visits with their child in the past six months, and number of pediatric primary care visits in the past 6 months.

Both surveys were created using Google Forms, from which the responses could be exported to a spreadsheet for subsequent statistical analysis. Through the link to the form participants could access information on the study (study objective, justification, what the study participation consist and the applicability of the results, i.e. the intention to develop an error reporting system based on the study results), the informed consent form and the survey questions. Pediatricians were invited to participate in this study by email through the contact networks of the research team. The invitation for parents of children under 14 was disseminated on social media. The pediatrician survey was launched in November 2019 and closed in March 2020; the family survey was launched in April 2020 and closed in October 2020.

### Data analysis

Answers to open-ended questions were coded and analyzed using the thematic analysis with a deductive approach (number of spontaneous comments from the participants).

We performed a descriptive analysis, obtaining means and standard deviations of the quantitative variables; and frequencies of the qualitative variables. In order to compare the responses to questions that featured in both surveys (pediatricians and parents /caregivers), we applied the Chi-square statistic to contrast the frequencies of qualitative variables and multivariate techniques to measure different associations. *P* values below 0.05 were considered statistically significant. The statistical analysis was carried out using RStudio version 1.3.1093.

## Results

### Pediatrician survey

Our study included 182 pediatricians, of whom 168 (92.3%) lived in the Valencian Community, and 170 (93.4%) worked in primary care (Table 1).

**Table 1:** Characteristics of participating pediatricians (*n* = 182)

Characteristics	
Sex, n (%)	
Women	127 (69.8)
Men	48 (26.4)
No response	7 (3.8)
Age (years), n (%)	
25–40	61 (33.5)
41–55	67 (36.8)
56–90	51 (28.0)
No response	3 (1.6)
Years of experience in pediatrics, mean (SD)	21.2 (12.8)
Specialty	
Surgery	1 (0.5)
Neuropediatrics	4 (2.2)
General and primary care pediatrics	170 (90.7)
Hospital pediatrics	2 (1.1)
Pediatric resident	1 (0.5)
Emergency medicine	1 (0.5)
No response	3 (1.6)
Care setting, n (%)	
Hospital	9 (4.9)
Primary care	170 (93.4)
No response	3 (1.6)
Spanish autonomous community, n (%)	
Andalusia	2 (1.1)
Aragon	1 (0.5)
Castilla la Mancha	1 (0.5)
Catalonia	2 (1.1)
Valencian Community	168 (92.3)
Madrid	3 (1.6)
Basque Country	1 (0.5)
No response	4 (2.2)

#### Difficulties in caring for children

**Fig. 1:**
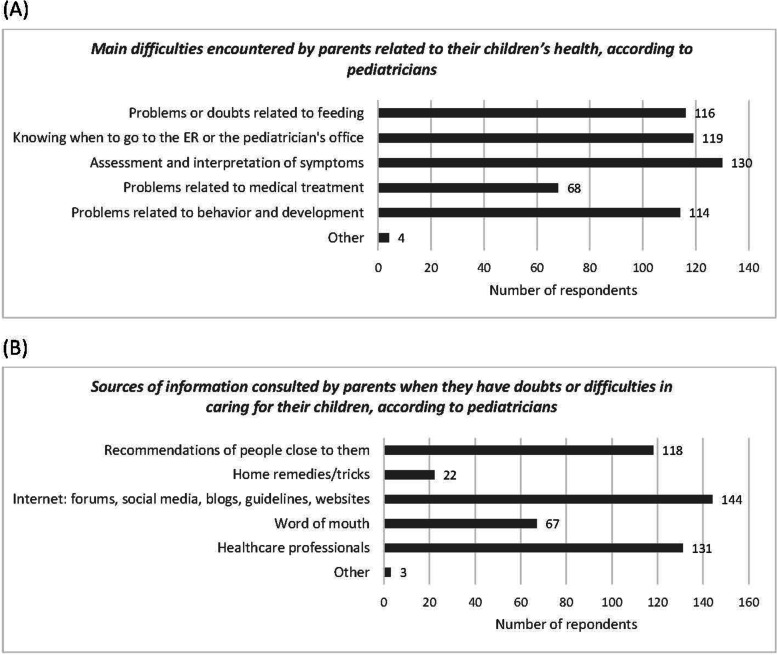
Graph of responses of pediatricians to the questions about **A** main difficulties encountered by parents related to their children health and **B** sources of information consulted by parents when they have doubts or difficulties in caring for their children

The responses to the question on general difficulties faced by parents regarding the health of their children show that 62.6% of pediatricians (*n* = 114) did not consider medical treatment, i.e. all aspects related to the administration of a medication to a child, to be one of the main difficulties. Regarding the resolution of doubts or difficulties regarding the care of children, 79.1% (*n* = 144) of pediatricians believed that parents consulted the internet (Fig. 1). The difference in frequency between this and the remaining options was statistically significant (*p* < 0.05).

#### Medication errors at home

**Fig. 2:**
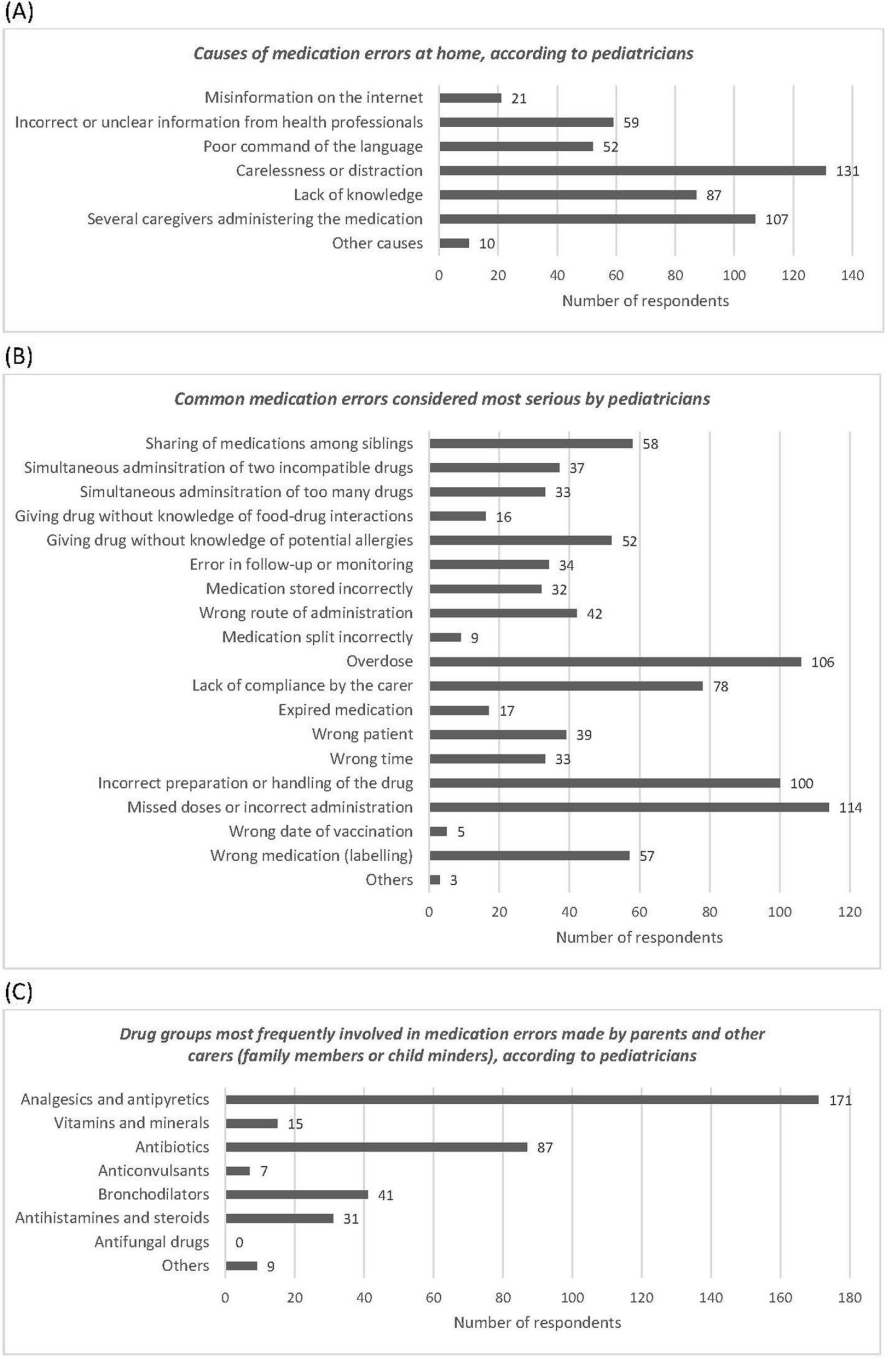
Graph of responses of pediatricians to the questions about **A** causes of medication errors at home, **B** common medication errors considered most serious and **C** drug groups most frequently involved in medication errors made by parents and other carers

According to the pediatricians who completed the survey, the most common causes of medication errors at home are carelessness or distraction (72.0%), having several caregivers administering medication (58.8%) and lack of knowledge (47.8%). The respondents also suggested other causes: presentation of the same drug at different concentrations, unsuitable advice from pharmacists, inappropriate prescriptions written in the ER by non-pediatricians, unclear patient information leaflets, polypharmacy, complexity of drug preparation, conflicting dosage information between patient information leaflets and prescriptions, overprotection of children, and some dosing devices. More than half of respondents considered that missed doses or incorrect administration (62.6%, *n* = 114), overdose (58.2%, *n* = 106) and incorrect preparation or handling of the drug (54.9%, *n* = 100) were severe common medication errors. Analgesics and antipyretics were considered the type of drug most commonly involved in these errors (94.0%, *n* = 171), with a significant difference between this and the remaining options (*p* < 0.05) (Fig. 2).

#### Error prevention program

**Fig. 3:**
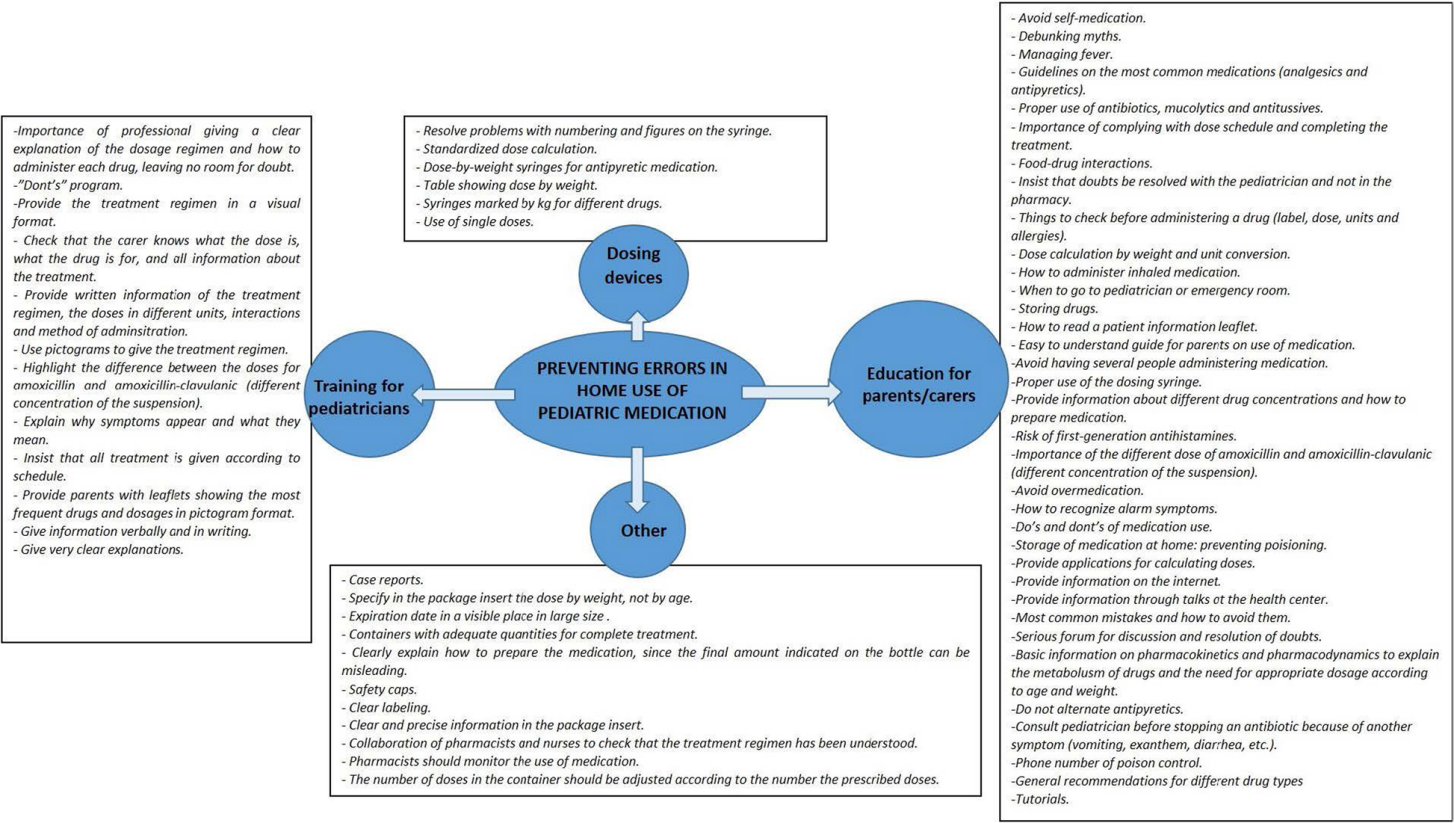
Areas of improvement to be included in a program aimed at preventing possible pediatric medication errors at home

**Fig. 4:**
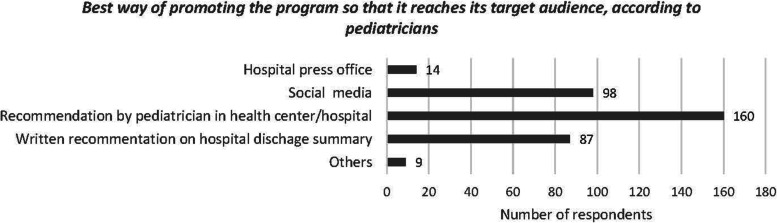
Graph of responses of pediatricians to the question about best way of promoting a program aimed at preventing possible pediatric medication errors at home

When the pediatricians were asked what should be included in a program aimed at preventing possible pediatric medication errors at home, the responses of pediatricians were grouped according to the following areas of improvement: knowledge of parents and caregivers; information provided by pediatricians when prescribing medication; dosing devices; and other (Fig. 3). Moreover, 95.1% (*n* = 173) of pediatricians surveyed stated that they would recommend an intervention program to their patients to prevent any pediatric medication errors at home. Most respondents considered that the best way of disseminating such a program would be at the pediatrician’s office (87.9%, *n* = 160) and through social media (53.8%, *n* = 98) (Fig. 4).

### Family survey

In total, 194 parents or caregivers responded to the survey, of whom 153 (78.9%) were women,159 (82.0%) lived in the Valencian Community, and 108 (55.7%) had a university education (Table 2).

**Table 2:** Characteristics of participating parents (*n* = 194)

Characteristics	
Sex, n (%)	
Women	153 (78.9)
Men	40 (20.6)
No response	1 (0.5)
Age in years, mean (SD)	40.6 (6.2)
Marital status, n (%)	
Married/in registered partnership	153 (78.9)
Divorced	7 (3.6)
In a relationship (not married)	21 (10.8)
Separated	4 (2.1)
Single	8 (4.1)
No response	1 (0.5)
Educational attainment, n (%)	
High school diploma	9 (4.6)
Vocational training	52 (26.8)
Compulsory education (until age 16)	23 (11.9)
Private degree studies	1 (0.5)
Bachelor’s degree	60 (30.9)
Master’s/Doctorate degree	48 (24.7)
No response	1 (0.5)
Number of children, n (%)	
1	85 (43.8)
2	87 (44.8)
3	18 (9.3)
4	3 (1.5)
No response	1 (0.5)
No. of emergency room visits with a child in past 6 months, n (%)	
0	114 (58.8)
1	55 (28.4)
2	9 (4.6)
3	7 (3.6)
4	5 (2.6)
5	2 (1.0)
10	1 (0.5)
No response	1 (0.5)
No. of pediatric primary care visits in past 6 months, n (%)	
0	61 (31.4)
1	65 (33.5)
2	25 (12.9)
3	17 (8.8)
4	13 (6.7)
6	3 (1.5)
8	2 (1.0)
9	3 (1.5)
10	3 (1.5)
30	1 (0.5)
No response	1 (0.5)
Spanish autonomous community, n (%)	
Andalusia	3 (1.5)
Aragon	1 (0.5)
Asturias	1 (0.5)
Castilla la Mancha	1 (0.5)
Catalonia	7 (3.6)
Valencian Community	159 (82.0)
Extremadura	2 (1.0)
Madrid	4 (2.1)
Region of Murcia	15 (7.7)
No response	1 (0.5)

#### Difficulties in caring for children

**Fig. 5:**
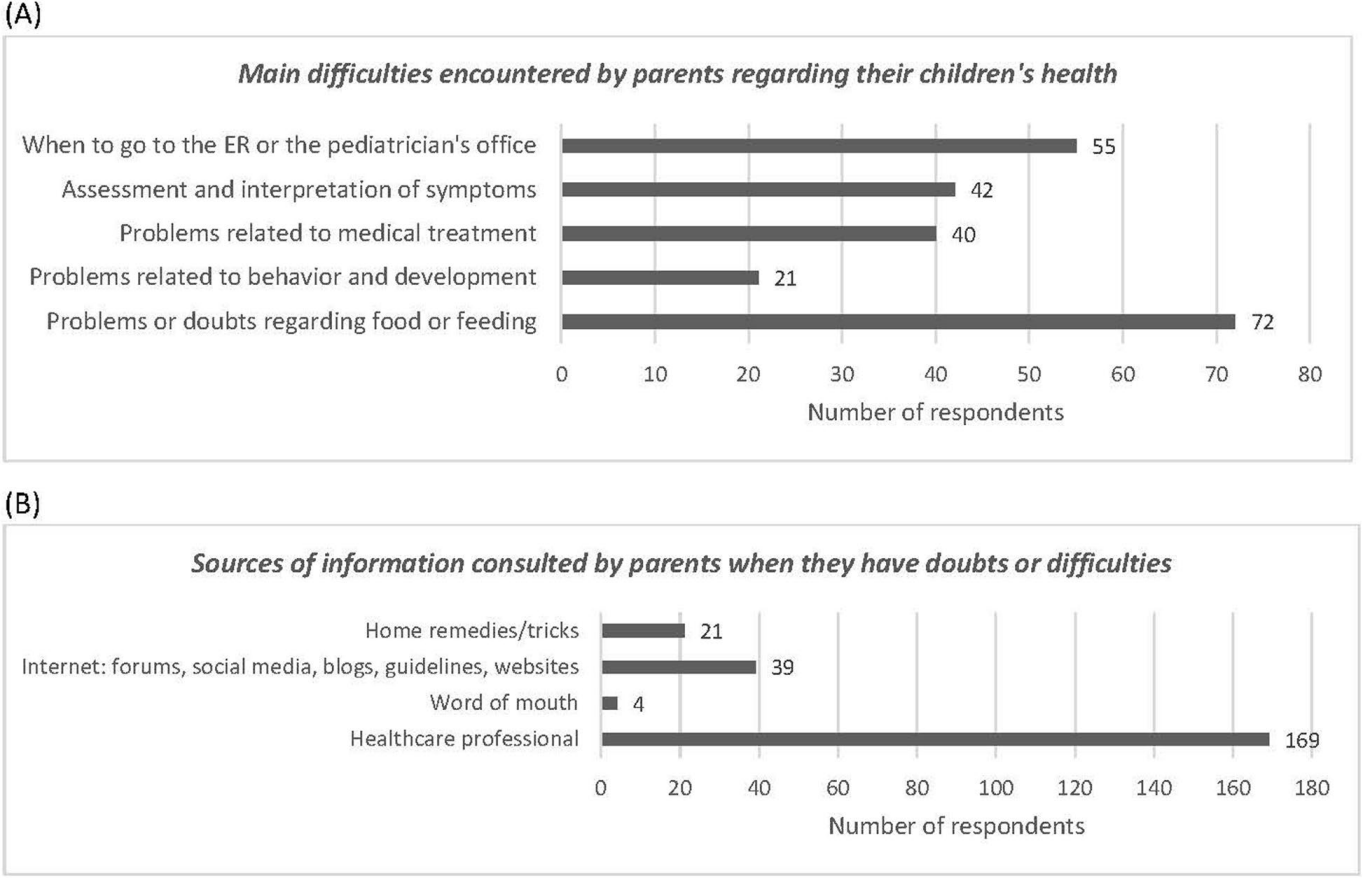
Graph of responses of parents/caregivers to the questions about **A** main difficulties encountered by parents related to their children health and **B** sources of information consulted by parents when they have doubts or difficulties in caring for their children

Of all respondents to the family survey, 37.1% (*n* = 72) reported at least one difficulty regarding food or feeding, 10.9% (*n* = 21) described a psychomotor difficulty, 20.7% (*n* = 40) referred to a difficulty with medical treatment, and 28.5% (*n* = 55) said they had difficulty knowing when to go to the ER or to the pediatrician (Fig. 5). Regarding difficulties related to medical treatment, the respondents mentioned the following aspects: calculation of doses, treating infants, ineffective treatment, refusal of the child to take the medicine, adverse effects, adherence to the dosing schedule, scarce availability of the drug in pharmacies, vomiting after administration, and inhaled drugs. Just over one fifth of families (21.1%, *n* = 41) thought that medical treatment was one of the main difficulties they had faced with respect to their children’s health, while the option ‘assessment and interpretation of symptoms’ was selected by significantly more families than were the remaining options (*p* < 0.05) (Fig. 5). Most families (87.1%, *n* = 169) said they consulted a healthcare professional when in doubt (*p* < 0.05) (Fig. 5).

#### Medication errors at home

**Fig. 6:**
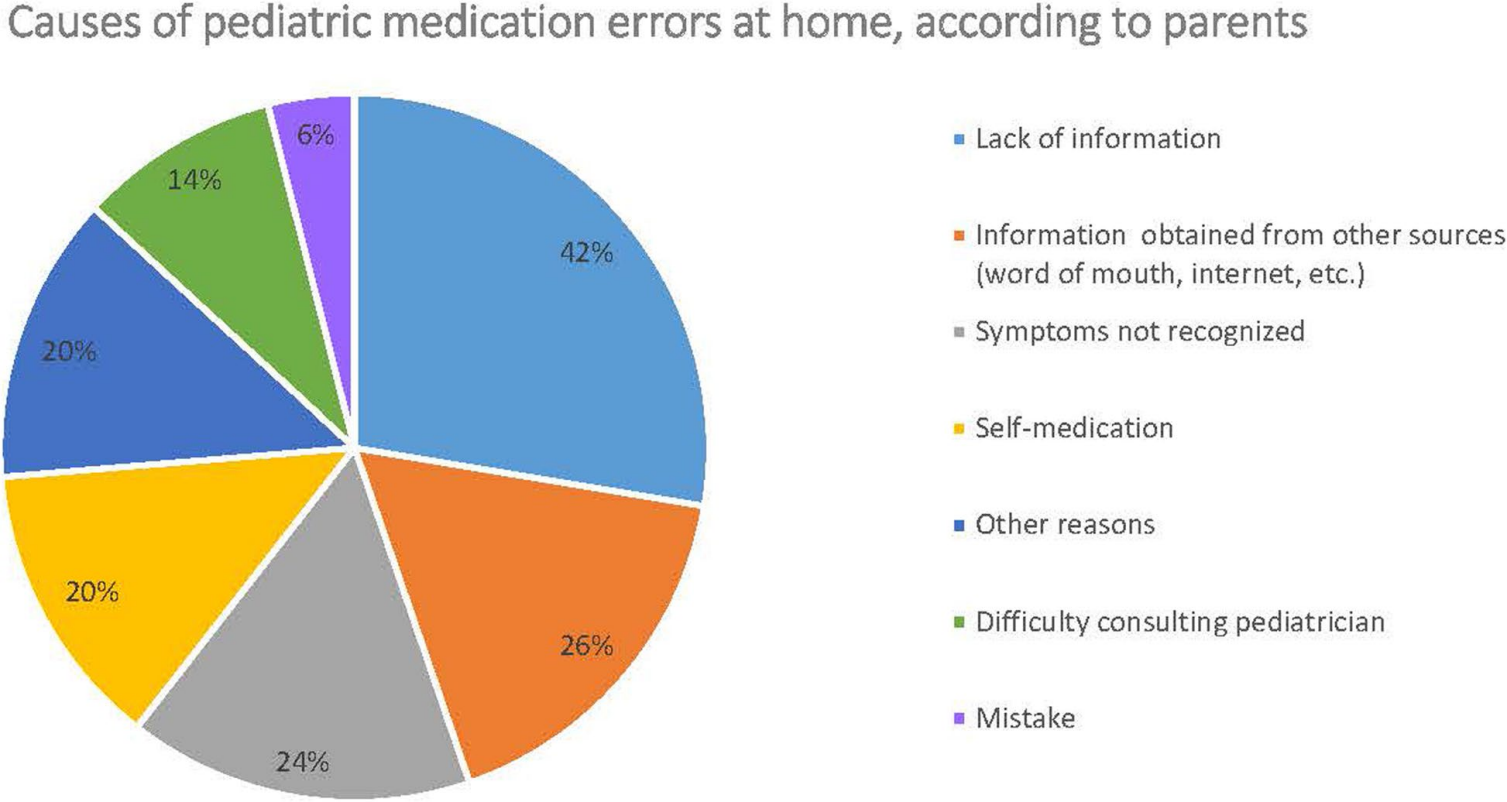
Causes of medication errors at home according to parents/caregivers

**Fig. 7:**
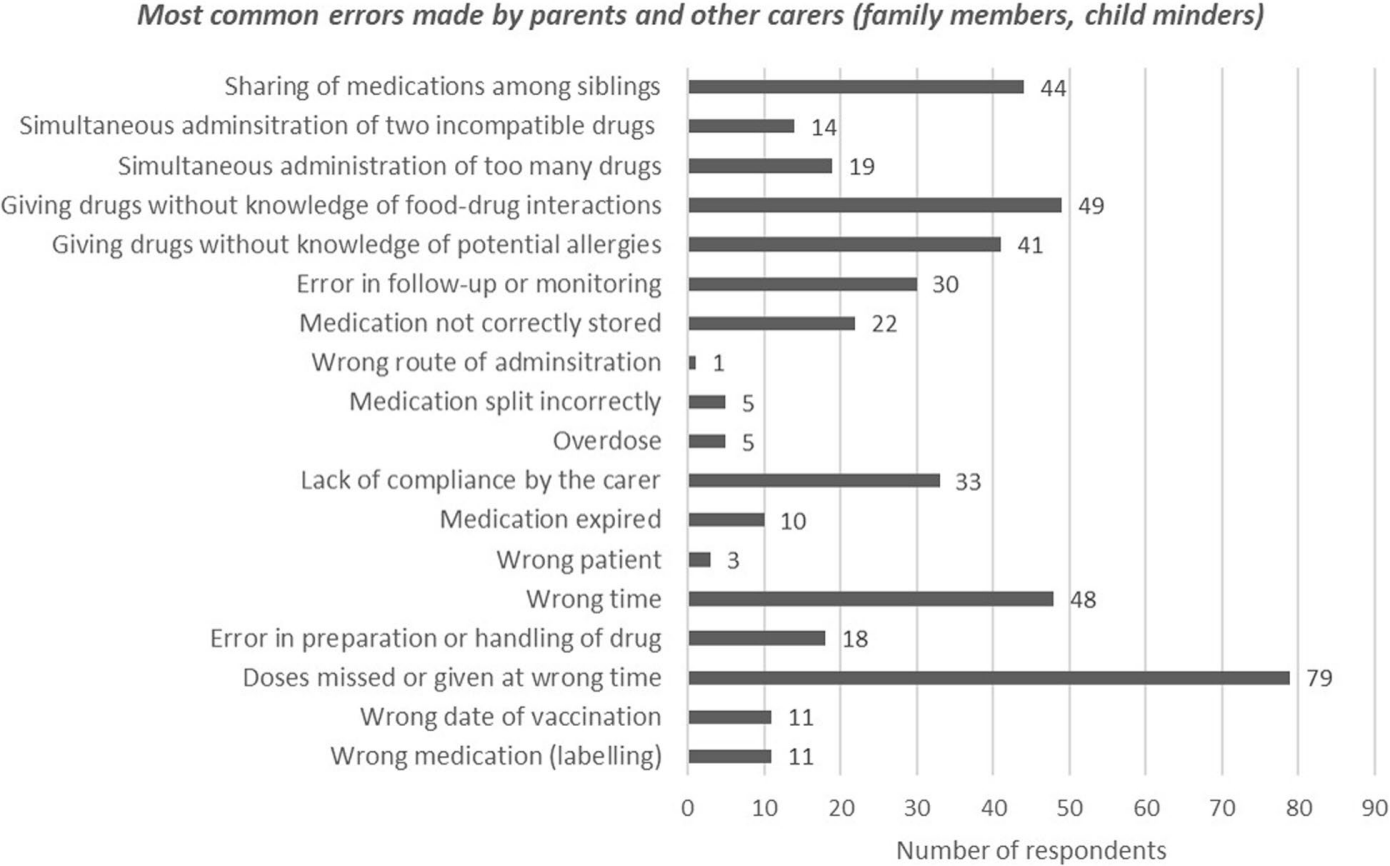
Graph of responses of parents/caregivers to the questions about most common errors made by parents and other carers (family members, child minders)

When families were asked about causes of pediatric medications errors, they referred to insufficient provision of information to parents, unreliable sources of information, self-medication, and the difficulty of consulting a pediatrician (Fig. 6). Sixty respondents did not answer this question. Regarding the types of medication errors that families consider most common, three options were selected with significantly higher frequency than the rest (*p* < 0.05): missed doses or incorrect administration (40.7%, *n* = 79), giving drugs without knowledge of food-drug interactions and (25.5%, *n* = 49) and wrong time (24.7%, *n* = 48) (Fig. 7).

#### Error prevention program

The surveyed families considered that a platform aimed at preventing medication errors in the pediatric population should include the following aspects: recognizing and managing symptoms; methods of drug administration; recommendations for correctly administering medication to children; antibiotics; how to calculate and convert doses; drug compatibility; vaccines; recommendations for remembering dosing schedules; severity of side effects; consequences of medication errors; when to go to the pediatrician or emergency room; allergies; homeopathic medication; diet; vitamins; child development; resolution of doubts; answers to frequently asked questions about medication; real cases of medication errors; myths and facts; first aid; guidelines for parents; recommendations for keeping children healthy; dental health; physical activity; stimulation exercises; sleep; and child psychology and emotions. Respondents said they would prefer this information to be organized by age (51.3%, *n* = 99) or by symptoms (43.0%, *n* = 83). Half of the surveyed families (50.5%, *n* = 98) considered that the most suitable format for the program would be a mobile application, and 60.2% (*n* = 117) thought that the best way for parents to discover it would be through their family pediatrician.

### Differences between pediatricians and families

When we compared the responses to questions that featured in both surveys (‘the main difficulties encountered by parents in caring for their children’ and ‘sources of information that parents consult when they have doubts or difficulties in caring for their children’), we found that pediatricians were more likely than families to believe that the main difficulties faced by parents in caring for their children were related to food or feeding (64.9% vs. 28.4%), medical treatments (36.1% vs. 21.1%) and knowing when to go to the emergency room or pediatrician (64.9% vs. 33.5%) (*p* < 0.05). In addition, significantly more pediatricians than families believed that parents’ main source of information for resolving doubts was word of mouth (35.1% vs. 2.1%), the internet (78.5% vs. 20.1%) and home remedies (65.4% vs. 10.8%); while significantly fewer pediatricians than families (69.6% vs. 87.1%) thought that parents would consult a health professional for this purpose (*p* < 0.05).

## Discussion

The results of this study reveal the perceptions and attitudes of pediatricians and parents with regard to pediatric medication errors in the home setting. Firstly, a series of open questions are asked of parents to find out about the problem situations that occur with their children and how important spontaneous medication errors are for them, followed by a focus on what medication errors in paediatrics are and the importance of designing home-based reporting systems to prevent them. Problems with food/feeding and knowing when to go to the pediatrician were considered more important than problems related to medical treatment. While most pediatricians thought that parents used the internet as their main source of information for resolving doubts related to their children’s health, most parents said they consulted a healthcare professional in such cases. Regarding errors in the home use of pediatric medication, parents’ and caregivers’ lack of knowledge was one of the causes most frequently mentioned by both pediatricians and parents. Most pediatricians said they would recommend a program designed to prevent these errors to the families of their patients.

Among the main difficulties encountered by families in caring for their children is the lack of understanding the nature of the disease and how to manage it properly, difficulty administering medications to their children, either due to dosage problems, storage problems, or problems remembering administration, financial problems or lack of social support [14–16]. Although our results suggest that managing medical treatment is not recognized as a major difficulty. According to families’ perceptions, there are other childcare issues that also require attention, previous studies have found that parents and caregivers make dosing errors [17]; prepare medications incorrectly [9]; medicate their children without prior recommendation from a pediatrician, especially to treat fever or coughs and colds [18]; and have difficulty identifying and managing symptoms (e.g., there are still misconceptions about fever) [19].

Pediatricians and families seemed to agree that medication errors at home are due to lack of knowledge and lack of information. Previous studies confirmed that parents and caregivers of pediatric patients made errors because they did not understand the treatment instructions [9, 20] or did not know how to convert between units of measurement [21] or how to prepare the medicine [9]. Although dosing errors constitute the type of pediatric medication error most studied and discussed in the scientific literature, only a minority of the families surveyed for this study thought that they made this kind of error when administering medicine to their children. The pediatricians agreed with the published literature [6] in considering that analgesics and antipyretics constitute the pharmacological group most frequently involved in medication errors at home.

Concerning the sources of information most frequently consulted by families to resolve doubts about their children’s health or medical treatments, a 2011 study conducted in Spain indicated that a high proportion of families consulted the internet [22]. However, the families who participated in this study said they were more likely to consult a healthcare professional.

As far as we know, there are currently no systems in place for reporting errors in the home use of medication. The pediatricians surveyed for this study said they would recommend an informative intervention program to the families of their patients to prevent possible medication errors. Similarly, the families said they would like to have access to all kinds of information on the care of their children, and would welcome a reliable web site where they could find answers and report errors.

Developing future strategies to prevent pediatric medication errors requires the input of pediatricians and parents or caregivers. The results of this study could therefore help pediatricians, clinical directors, pharmacists and health policy makers to ensure comprehensive patient care. The survey responses could also be used to develop preventive measures in the pediatric primary care or community pharmacy setting, or to develop error notification systems for parents and caregivers. This study takes the forefront of the new strategies implemented by national health systems in different countries, where caregivers describe their information needs in order to provide tools for better management of home care, particularly in relation to medication. Providing strategies to prevent medication errors and learning from mistakes made at home appear to be new challenges to address [23].

The main limitations of this study include the use of an ad-hoc survey to collect the opinion of study participants without information on the validity and reliability of the questionnaire, and the potential bias resulting from nonprobability sampling. Indeed, most of our respondents lived in the Valencian Community, and our results therefore cannot be generalized to the whole population. Because most of the questions in the family survey were open, some participants misinterpreted questions or were unable to answer them. This may be due to false beliefs regarding possible medication errors. Finally, to ensure data anonymity, respondents were not asked to provide personal data, meaning the same person could complete the survey more than once. To prevent this, however, we checked respondents’ IP addresses.

## Conclusion

Most pediatricians and families believe that following medical treatment is not among the main difficulties faced by parents in caring for their children. To resolve doubts related to their children’s health, most parents say that they consult a healthcare professional, while most pediatricians think that parents consult the internet. Pediatricians and families alike believe that parents’ and caregivers’ lack of knowledge is a main cause of pediatric medication errors. This study has been able to describe the differences between the perceptions of families and paediatricians regarding the different problem situations that can be found in the home. Having this classification of problem situations from both points of view and the importance of medication errors and the sources of information that should be consulted in case the information is needed are fundamental elements when building a reporting system. The reporting of the error or problematic situation can contribute to generating recommendations so that families learn to better manage this type of situation, always bearing in mind that in case of doubt, the healthcare professional is the point of reference, as this study has also found.

## Supplementary Information

**Additional file 1: Supplementary Document 1.** Pediatrician questionnaire: safer use of medication in pediatric patients at home. **Supplementary document 2.** Family questionnaire: safe use of medication. **Supplementary Document 3.** Aspects to include in a pediatric prevention program, according to pediatricians.

## Data Availability

The datasets used and/or analysed during the current study available from the corresponding author on reasonable request.

## Abbreviation

ER: Emergency room
