# Supplementary material for: Perceptions and attitudes of pediatricians and families with regard to pediatric medication errors at home
Source: BMC Pediatr. 2023 Jul 31;23:380. doi: 10.1186/s12887-023-04106-x (PMC10391897; doi:10.1186/s12887-023-04106-x)
Supplement: Supplementary file 1 — Additional file 1: Supplementary Document 1. Pediatrician questionnaire: safer use of medication in pediatric patients at home. Supplementary document 2. Family questionnaire: safe use of medication. Supplementary Document 3. Aspects to include in a pediatric prevention program, according to pediatricians. [file 12887_2023_4106_MOESM1_ESM.docx]

Supplementary Material

# Supplementary Document 1.

# Pediatrician questionnaire: safer use of medication in pediatric patients at home

1. Do you agree with the following classification of the pediatric population?
2. Neonates
3. Infants
4. Preschoolers up to 5 years
5. Schoolchildren from 6 to 12 years

- Yes
- No

If you answered No, can you explain why? (Open text question)

1. In your opinion, the main difficulties encountered by parents in caring for their children are related to
   1. Food or feeding
   2. Psychomotor learning
   3. Medical treatment
   4. Symptoms
   5. Knowing when to go to the emergency room or to the pediatrician
   6. Other (open text question)
2. In your opinion, which sources of information do parents consult when they have doubts or difficulties in caring for their children?
3. A healthcare professional
4. Word of mouth
5. Internet: forums, social media, blogs, guidelines, pediatric websites
6. Home remedies/tricks
7. Recommendations from people close to them
8. Other (open text question)
9. The second part of this questionnaire aims to know your opinion about pediatric medication errors at home. In your opinion, what are the causes of these errors?
10. Several caregivers administering medication
11. Lack of knowledge
12. Misinformation on the internet
13. Other causes (open question)
14. Of the following common medication errors that occur at home, which do you consider most serious? Please add any others that you feel are missing from the list.
15. Wrong medication (labeling)
16. Vaccination appointment outside the recommended age period
17. Missed doses or incorrect administration
18. Error in drug preparation or handling
19. Wrong time
20. Wrong patient
21. Expired medication
22. Lack of compliance by the caregiver
23. Overdose
24. Incorrect splitting of medication
25. Wrong route of administration
26. Incorrect storage of medication
27. Error in follow-up or monitoring
28. Giving drugs without knowledge of potential allergies
29. Giving drugs without knowledge of food-drug interactions
30. Administration of too many drugs at the same time
31. Administration of two incompatible drugs
32. Sharing of medication among siblings
33. Other errors that do not appear in the list (open question)
34. In your experience, what drugs are most commonly involved in pediatric medication errors made by parents or other caregivers (family members or child minders)?
35. Analgesics and antipyretics
36. Vitamins and minerals
37. Antibiotics
38. Anticonvulsants
39. Bronchodilators
40. Antihistamines and steroids
41. Antifungal drugs
42. Other (open question)
43. In the space below, please suggest ideas for the contents of an intervention program aimed at preventing pediatric medication errors at home. (Open question)
44. What do you think is the best way to disseminate the intervention program so that it reaches its target audience?
45. A written recommendation on hospital discharge summaries
46. Recommendation by pediatricians in health centers and hospitals
47. Social media
48. Hospital press office
49. Other (open question)
50. Finally, could you suggest possible names for this intervention program so that it can be recommended and remembered by healthcare professionals and parents who use it? (Open question)
51. Would you recommend this type of platform to your patients?

- Yes
- No

If you answered no, can you explain why? (Open text question)

**Supplementary document 2.**

**Family questionnaire: safe use of medication**

1. What difficulties have you encountered so far in caring for your children with regard to food or feeding? (Open question)
2. What difficulties have you encountered so far in caring for your children with regard to psychomotor learning? (Open question)
3. What difficulties have you encountered so far in caring for your children with regard to medical treatment? (Open question)
4. What difficulties have you encountered so far in caring for your children with regard to symptoms? (Open question)
5. What difficulties have you encountered so far in caring for your children with regard to knowing when to go to the emergency room or to the pediatrician? (Open question)
6. When you have these difficulties or doubts, where or who do you consult for advice?
   1. A healthcare professional
   2. Word of mouth
   3. Internet: forums, social media, blogs, guidelines, pediatric websites
   4. Home remedies or recommendations from people you are close to
7. According to your comments, and according to the scientific literature, errors in the home use of children’s medication are quite common. Why do you think these medication errors occur? (Open question)
8. In your experience, what types of error are most common?
   1. Wrong medication (labeling)
   2. Vaccination on wrong dates
   3. Missed doses or incorrect administration
   4. Error in drug preparation or handling
   5. Wrong time
   6. Wrong patient
   7. Expired medication
   8. Lack of compliance by the caregiver
   9. Overdose
   10. Incorrect splitting of medication
   11. Wrong route of administration
   12. Incorrect storage of medication
   13. Error in follow-up or monitoring
   14. Giving drugs without knowledge of potential allergies
   15. Giving drugs without knowledge of food-drug interactions
   16. Administration of too many drugs at the same time
   17. Administration of two incompatible drugs
   18. Sharing of medication among siblings
9. In your opinion, which drugs are most commonly involved in errors?
   - 1. Analgesics and antipyretics
     2. Vitamins and minerals
     3. Antibiotics
     4. Anticonvulsants
     5. Bronchodilators
     6. Antihistamines and steroids
     7. Antifungal drugs
10. What content would you like to see included in the platform? (Open question)
11. In what format? (Open question)
12. If you had any questions or concerns, how long would give professionals to answer? (Open question)
13. What would be the best way for this platform to reach parents? (Open question)
14. Would you like to see only the information relevant to the age group of your child, or information for all ages? (Open question)

**Supplementary Document 3.**

**Aspects to include in a pediatric prevention program, according to pediatricians.**

Regarding dosing devices

- Resolve problems with numbering and figures on syringes
- Standardized dose calculation
- Dose-by-weight syringes for antipyretic medication
- Table showing dose by weight
- Syringes marked by kg for different drugs
- Use of single doses

Education for parents/caregivers

- Dangers of self-medication
- Common myths
- Managing fever
- Guidelines on the most common medications (analgesics and antipyretics)
- Proper use of antibiotics, mucolytics and antitussives
- Importance of complying with dose schedule and completing the treatment.
- Food-drug interactions
- Importance of resolving doubts with the pediatrician and not in the pharmacy
- What to check before administering a drug (label, dose, units and allergies)
- Dose calculation by weight and unit conversion.
- How to administer inhaled medication
- When to go to pediatrician or emergency room
- Storing drugs
- How to read a patient information leaflet
- Straightforward guide for parents on use of medication
- Dangers of having several caregivers administering the medication
- How to use the dosing syringe correctly
- Information on different drug concentrations and how to prepare medication
- Risk of first-generation antihistamines
- Dosage of amoxicillin versus amoxicillin-clavulanic (different concentration of the suspension)
- Importance of avoiding overmedication
- How to recognize alarm symptoms
- Dos and don’ts of medication use
- How to safely store medication at home to avoid poisoning
- Most common mistakes and how to avoid them.
- Basic information on pharmacokinetics and pharmacodynamics to explain drug metabolism and the need for appropriate dosage according to age and weight
- Danger of alternating antipyretics
- Importance of consulting a pediatrician before stopping an antibiotic because of another symptom (vomiting, exanthem, diarrhea, etc.)
- Phone number of poison control center
- General recommendations for different drug types
- Tools/resources to provide to parents
  - Applications for calculating doses
  - Information on the internet
  - Information through talks at the health center
  - Serious forum for discussion and resolution of doubts
  - Tutorials

Recommendations for pediatricians

- Give clear explanations of the dosage regimen and how to administer each drug, leaving no room for doubt
- “Don’ts” program
- Provide the treatment regimen in a visual format
- Check that the caregiver knows what the dose is, what the drug is for, and all information about the treatment
- Provide written information of the treatment regimen, the doses in different units, interactions, and method of administration
- Use pictograms to explain the treatment regimen
- Highlight the difference between the doses for amoxicillin and amoxicillin-clavulanic (different concentration of the suspension)
- Explain why symptoms appear and what they mean
- Insist that all treatment is given according to the schedule
- Provide parents with leaflets showing the most frequent drugs and dosages in pictogram format
- Give information verbally and in writing
- Give clear explanations

Miscellaneous

- Case reports
- Dose by weight (not by age) specified in the patient information leaflet
- Expiration date in a visible place in large size
- Containers with adequate quantities for complete treatment
- Clear explanation of how to prepare the medication, as the final amount indicated on the bottle can be misleading
- Safety caps
- Clear labeling
- Clear and precise information in the patient information leaflet
- Collaboration of pharmacists and nurses to ensure that the treatment regimen has been understood.
- Monitoring of medication use by pharmacists
- Number of doses in the container adjusted according to number of prescribed doses
